# Advancing the study of life and death education: theoretical framework and research inquiries for further development

**DOI:** 10.3389/fpsyg.2023.1212223

**Published:** 2023-07-27

**Authors:** Huy P. Phan, Si-Chi Chen, Bing H. Ngu, Chao-Sheng Hsu

**Affiliations:** ^1^School of Education, University of New England, Armidale, NSW, Australia; ^2^Department of Education, National Taipei University of Education, Taipei, Taiwan

**Keywords:** life and death, lifespan development, positive psychology, philosophical reflection, transformation, theoretical infusion, spiritual transcendence, esoteric experience

## Abstract

*Life and death education*, also known as *life education* and *death education*, is an interesting subject that may coincide with the subject of lifespan development. In brief, from our theoretical perspective, which guides our teaching and curriculum development, life education considers personal understanding of life functioning on a daily basis, whereas death education explores matters that are related to death and dying. For example, how can a social worker utilize his life knowledge, or life wisdom, to assist a relative to understand the intricate nature of death? In a similar vein, how can a senior citizen use her personal experience of Buddhist meditation practice to overcome a minor Covid setback? Central to our teaching practice is the premise of ‘active transformation’ (i.e., transforming life knowledge into positive practice) and the premise of ‘theoretical infusion’ (e.g., the infusion of a distinctive epistemological belief in the teaching of life) that would, in turn, help to enhance and facilitate deep, meaningful understanding of life and death. The purpose of the present article is for us to discuss a proposition of a theoretical-conceptual model, which depicts the ‘unification’ or integration of three major viewpoints of life and death: the *social viewpoint*, the *philosophical viewpoint*, and the *psychological viewpoint*. We theorize that unification of the three theoretical viewpoints may help provide grounding for effective teaching and holistic understanding of the subject contents of life and death. Such discourse, importantly, may also assist to advance the scope and complexity of the lifespan development subject. Finally, in addition to our theoretical-conceptual model of life and death, we propose three major research inquiries for development: the *meaning of situated mindset*, the *underlying nature of spiritual transcendence*, and *proposition of appropriate methodological accounts* for usage. Overall, then, we purport that our conceptual analysis and discussion overview, based on philosophical reflection, may serve to stimulate interest, intellectual curiosity, scholarly dialog, etc.

## 1. Understanding lifespan development, in brief

*Lifespan developmen*t (Peterson, 2004; Dacey et al., 2009; Sigelman et al., 2016), or *human development*, is an interesting field of study that offers theoretical insights into the *proactivity of human agency*. Human agency, in brief, is related to a person’s state of volition, freewill, and/or autonomy to make choices in life (e.g., a teenager’s determination to join a musical choir) and at the same time, to grow and flourish using different contextual conditions (Parsell et al., 2016; Bandura, 2018). By the same token, the study of lifespan development considers the importance of different types of personal development – for example, *cognitive development*, *emotional development*, *social development*, and *moral development* (e.g., moral development versus cognitive development) (Lickona, 1991; Dorough, 2011; Patanella, 2011). As part of lifespan development, say, cognitive growth or intellectual growth may help explain a child’s ability to appreciate the importance of moral values, which then would guide her to act and behave accordingly (Cowan et al., 1969; Lickona, 1991; Patanella, 2011). In a similar vein, social development (Sammons et al., 2008; Hasmath, 2014) espousing relevant social skills may assist a secondary school student to seek appropriate academic scaffolding for learning purposes.

It is interesting to note that the subject of lifespan development is often associated with the field of Education, especially in terms of preservice teacher education training. Many teacher education colleges and universities, for example, have various lifespan courses (e.g., *Child Development*) for pre-service teachers to undertake. It is reasoned that theoretical knowledge of the subject matter (e.g., in-depth understanding of morality)^1^ would help teacher education graduates and/or in-service teachers to understand their students better. For example, in-depth understanding of emotional development (Bazalgette et al., 2015; Ciarrochi et al., 2015) may assist newly graduates of teacher education and in-service teachers to appreciate the importance of student happiness, which plays a prominent role in the teaching and learning processes (Spice, 2011; Tabbodi et al., 2015). In a similar vein, personal understanding of ‘morality’ (Abel, 1989; Stanger et al., 2013) may enable in-service teachers to identify logical reasons for children’s dishonest and/or unethical behavioral patterns.

Indeed, existing literatures have shown that the scope of lifespan development (Peterson, 2004; Dacey et al., 2009; Sigelman et al., 2016) is relatively distinct, reflecting rigorous scientific knowledge and understanding of a person’s lifetime development (e.g., a child’s progression in moral development from elementary to secondary school) (Phan and Ngu, 2019). As such then, as researchers of lifespan development (e.g., cognitive development in academic contexts), we are interested to advance the scope of this subject by situating it within the framework of ‘life and death education’, also known as *life education* and *death education* (Huang, 2014; Tsai et al., 2020; Phan et al., 2020a,b). In other words, as a brief mentioning, we rationalize that the content of life and death may help to make a theoretical contribution to the scope lifespan development. For example, appreciating the ‘true meaning of life’ is somewhat personal and reflects a person’s cognitive maturity. Such appreciation for life and/or of life, in this sense, may help the person to perhaps approach life with sense of positivity and optimism, regardless of his perceived hardship, difficulty, etc. In a similar vein, what can we say about the subject of *death*? That the subject itself is negative and morbid, instilling a perceived sense of dread, angst, discomfort, uneasiness, etc.

## 2. Purpose and focus for consideration

The *purpose* of our conceptual analysis article is to advance the scope of lifespan development (Peterson, 2004; Dacey et al., 2009; Sigelman et al., 2016) by considering the importance of another related subject, known as life and death (Huang, 2014; Tsai et al., 2020; Phan et al., 2020a,b). The incorporation of life and death education may help to expand the complexities and subject contents of lifespan development (e.g., that philosophical understanding of death may help to highlight a teenager’s cognitive maturity). For example, in addition to the study of physical growth, cognitive intelligence, and/or the development of social skills, lifespan development may also consider the relevance and applicability of one’s philosophical reflection of his daily life functioning for different domains. This premise, in this sense, focuses on the central relevance of life, death, and its intimate relationship within a person’s lifespan.

By all account, there are different theoretical and/or cultural viewpoints pertaining to the teaching of life and death (Segal, 2004; Carrera, 2009; Picone, 2012; Reif et al., 2014; Joaquin, 2019). What is life and, likewise, what is death? Is there universal consensus (e.g., viewpoint, interpretation, inference, theoretical understanding, etc.) by which we all could agree upon in the teaching of life and/or of death? For example, in terms of differing viewpoints, the Buddhist viewpoint of death and dying (Masel et al., 2012) is different from the Islamic viewpoint, which Kristiansen and Sheikh (2012) state the following: “Life and death are believed by Muslims to be in accord with the will of Allah – the timing of death is therefore predetermined with a fixed term for each human being. Death marks the passing to the *Hereafter* – the ultimate destination. Our earthly life is considered a testing ground and our relations are trusts from Allah, which we are asked to treasure, but in doing so to remember that these are ultimately to be returned to Him” (p. 513–p. 514). Following on from this, Carrera’s (2009) article, titled ‘*Lovesickness and the therapy of desire: Aquinas, cancionero poetry, and Teresa of Avila’s ‘Muero porque no muero*’, captures an important viewpoint of death and dying – that from the literary legacy of Teresa of Ávila, also known as Saint Teresa of Jesus, a person may find life in death (e.g., ‘Muero porque no muero’, which translates to ‘I die because I do not die’). This aforementioned mentioning about death and dying is insightful as it emphasizes variations or differences in epistemologies, theoretical understandings, cultural beliefs, customary practices, etc.

In a similar vein, in relation to life education, personal belief in the ‘essence’ and/or the meaning of daily life functioning for any person for that matter (e.g., attaining financial wealth versus the seeking of self-actualization) may differ from one culture to that of another culture (Phan et al., 2021a,b). One of our reviewers earlier on provided his own interesting viewpoint of life and death education, drawing from personal understanding of Western epistemologies (Frankl, 1984; Carrera, 2009; Reif et al., 2014), which we would like to share. For example, the reviewer referred to the work of Frankl (1984) and his ideas of life functioning (e.g., what is the ultimate meaning of life?). According to Frankl (1984), as humans, we often look to those extreme life situations or margins that would test our inner strengths and characters. The ultimate test then, for all of us, is to find true meaning in our lives, regardless of social standing, health, financial wealth, etc. (e.g., “*Man can preserve a vestige of spiritual freedom, of independence of mind, even in such terrible conditions of psychic and physical stress*”).

**Figure 1:**
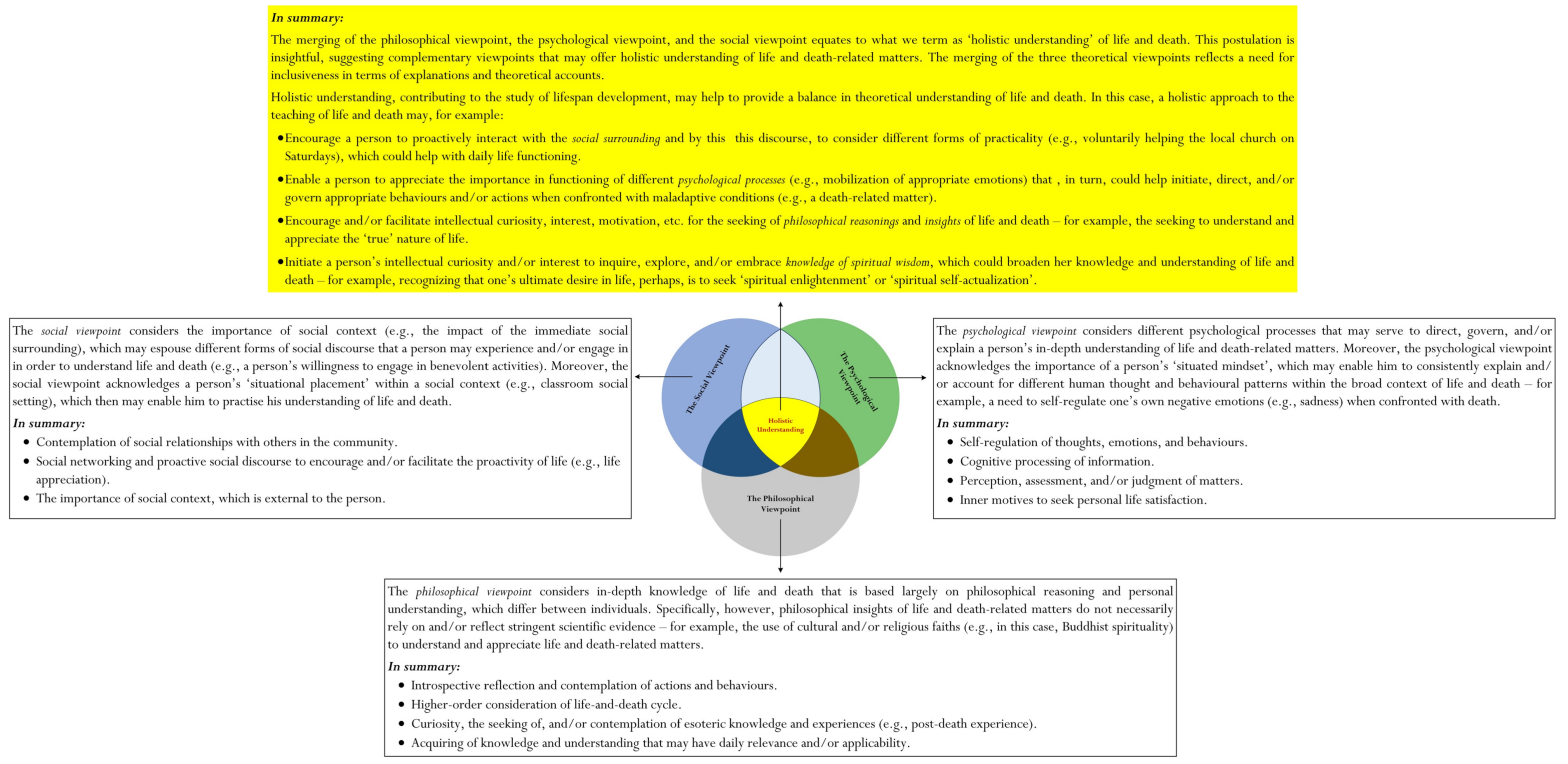
Merging of social viewpoint, philosophical viewpoint, and psychological viewpoint of life and death.

Our focus on life and death education (Huang, 2014; Tsai et al., 2020; Phan et al., 2020a,b) introduces readers to our personal teaching and research discourse. For reader interest, we have decided to discuss our teaching and research experiences with reference to *the sociocultural-learning context of Taiwan*. Taiwan, in brief, is relatively strong on her focus of quality teaching, innovative curriculum development, and applied practice of life and death education (Huang, 1993; Chen, 2013; Ministry of Education Taiwan, 2018; Phan et al., 2021a,b). From an early age, for example, Taiwanese children are exposed to this subject in some form. In a similar vein, in acknowledgment of individuals, families, and the community, as well as the importance of the subject itself, the Taiwanese Ministry of Education actually dedicated 2001 as the ‘Year of Life Education’ (Phan et al., 2020a,b). Moreover, we purport that theoretical overview and discussion of the Taiwanese sociocultural-learning context of life and death would help stimulate intellectual curiosity and offer grounding for us to undertake two major objectives:

▪ *Objective 1*: *To propose a theoretical framework*, *as shown in* Figure 1, *for further conceptualization and/or development* – ▪Our proposed theoretical framework considers three major approaches that may integrate, or unify, to provide holistic knowledge and understanding of life and death: the *Social approach*, the *Psychological approach*, and the *Philosophical approach*

▪ *Objective 2*: *To propose a conceptualization in research inquiries*, *based on the proposed theoretical framework*, *for advancement* – Complementing our proposed theoretical framework is a proposition of three interrelated inquiries for continuing research development: the importance of *a situated mindset*, one’s own possible *transcendence and esoteric experience,* and an *appropriate methodological account* for usage.

## 3. In brief: the importance of life and death

As a brief introduction, aside from research development, all of us have been and are involved in the teaching of both lifespan development (Peterson, 2004; Dacey et al., 2009; Sigelman et al., 2016) and life and death education (Chen, 2001, 2009; Tsai et al., 2018; Phan et al., 2021a,b; Lei et al., 2022). For example, the first author and the third author teach lifespan development and life and death education to both undergraduate and postgraduate students in Australia, whereas the second author and the fourth author, in contrast, teach life and death education to undergraduate and postgraduate students in Taiwan. As such, we complement each other in terms of our research and teaching experiences of the two subject areas, allowing us to progress and make meaningful contributions in terms of knowledge building, seeking new research frontiers, and establishing appropriate instructional methods for effective learning. For example, recently, our published article explored the extent to which one could use *positive psychology* and *positive education* (Seligman and Csíkszentmihályi, 2000; Seligman et al., 2009; Csíkszentmihályi, 2014) to gain in-depth understanding of life and death. We proposed that comparable characteristics of positive psychology (e.g., the importance of optimism and/or hope), for instance, may operate to help individuals in society cope with different forms of maladaptive life functioning (e.g., personal grief).

### 3.1. Life education: a brief overview

Our specific teaching experiences of life education to both undergraduate and postgraduate students reflect the perspective of Taiwanese Education (Chen, 2012; Ministry of Education Taiwan, 2018). The subject content of life, in brief, considers the importance of ‘life functioning’ for individuals and society, both short-term (e.g., a student’s social relationship with her peers as she works on a group assignment) and long-term (e.g., a graduate’s goals for the next five years for accomplishment). Life functioning, as the nomenclature connotes, relates to a person’s functioning on a daily basis (i.e., how is he/she going at the moment for a particular context?) for different domains and/or courses of action (e.g., the importance of social functioning versus academic functioning). In this sense, from our point of view, positive and/or proactive life functioning, in general, is a central tenet of life itself. Understanding the meaning and/or the nature of life functioning, in general, would help a person to have differing opinions, interpretations, viewpoints, etc. about daily life complexities (e.g., one’s financial struggle and personal hardship as a result of the Covid-19 pandemic).

Our teaching of life education is philosophical and open-ended, seeking to understand the *proactivity of life functioning*. What can a parent do to ensure that his children have self-fulfilling life experiences, for example, is an important question that resonates with our teaching of life education. In this analysis, our teaching of life education establishes and stipulates one important premise for development – namely:

The ‘transformation’ of *life knowledge*, or life wisdom (Sternberg and Glück, 2019, 2021), into positive *daily practice*, which in turn would reflect a person’s *consciousness*, *love*, and *care* for another person (e.g., a parent’s transformation of his life knowledge to help his children) (Phan et al., 2020a,b, 2021a,b).

The aforementioned premise of active transformation of life knowledge, or life wisdom, into daily practice (i.e., life wisdom → positive life practice) is an important aspect, which we focus on in our teaching of life and death. Central to this premise of transformation is the viewpoint, or the belief, that life knowledge, not necessarily academic *per se* (e.g., knowledge in Calculus), may be utilized appropriately for the purpose of effective life functioning. In other words, from our point of view, knowledge *per se* is a valuable investment and/or a commodity that may produce positive yields to help facilitate positive life and death experiences. For example, how can a senior citizen use his knowledge and understanding of ‘walking meditation’ (Phan et al., 2021a,b) to assist him with the Covid pandemic? In a similar vein, how can a teenager’s knowledge and understanding of Freud’s theory of moral conscience (Jones, 1966; Abel, 1989) help others in the neighborhood? Our teaching of death, as detailed next, also emphasizes the importance of active transformation of life knowledge.

Aside from the importance of active transformation of life knowledge, or life wisdom, we also incorporate different *religious faiths*, *cultural beliefs* and *epistemologies*, and *customary practices* in our teaching, as this practice would help facilitate appreciation and meaningful understanding of the subject matter. As a point for consideration, we use the term ‘theoretical infusion’ to denote and/or to reflect our practice of incorporation (e.g., the notion of ‘spiritual infusion’ in life teaching). For example, for the Taiwanese sociocultural-learning context, both the second author and the fourth author theoretically ‘infuse’ Buddhism (Yeshe and Rinpoche, 1976; Sheng Yen, 2010), Taoism (Saso, 1970; Rahul, 2018), and Confucianism (Yao, 2000; Havens, 2013) in their teaching of life and death. In relation to Buddhist philosophy, say, our teaching of life to students would highlight the importance of perceived positive ‘Buddha-like’ (佛系) qualities and/or characteristics, such as *ethical morality*, *truthfulness*, *equanimity*, and *kindness* (Chattopadhyay, 2022). From our point of view, attainment of ethical morality, truthfulness, equanimity, etc. may help a person to understand and to appreciate both the ‘ethics’ and the ‘esthetic nature’ of life. In a similar vein, our teaching of life education also infuses the philosophical premise of Confucianism (Yao, 2000; Havens, 2013). For example, we contend that good practice of life functioning entails a person’s reverence for the practice of ‘filial piety’ (Chow and Chu, 2007; Chen and Ho, 2012), such as her fulfillment of dutiful obligations (e.g., doing well academically at school), respecting elders and family members, helping others in society, etc.

By all account, we acknowledge that there may well be other theoretical approaches to the teaching of life education (Chen, 2009; Tsai et al., 2018; Phan et al., 2021a,b). As such, we contend that our teaching approach, somewhat philosophical, is not exclusive in nature. What is of significance, however, is that our teaching approach to life and death emphasizes the importance of theoretical infusion (e.g., spiritual infusion). That perhaps, to enhance teaching practice and theoretical understanding of life and death, we consider ‘infusing’ our approach with different contextual matters (e.g., the infusion or incorporation of Buddhist philosophy).

### 3.2. Death education: a brief overview

Our specific teaching experiences of death education, similar to that of life education, is philosophical and emphasizes the importance of theoretical infusion. Death, in its simplistic term, is defined as the ceasing of life of a living organism in the physical world (Phan et al., 2021a,b). The subject of death education, also known as *thanatology* (Meagher and Balk, 2013; Chapple et al., 2017), is not new and is relatively universal in terms of teaching and research development, despite variations in methodological designs, instructional approaches, epistemological and cultural beliefs, etc. For example, Tibetans (Prude, 2019), Indigenous Fijians,^2^ and Vietnamese (Son and Nga, 2019) have distinct cultural beliefs and viewpoints about death and dying and such differences, from our point of view, serve to accentuate the importance of ‘cultural specificity’.

We contend that the ceasing of life, or a person’s death, may come to espouse a perceived sense of distress, grief, despair, suffering, etc. One’s journey toward death or the ‘finalization of life’, from our experiences, is individualized and personal. Some, for example, may fear, suffer, and/or experience a state of angst and depression, whereas others may come to embrace and accept that the end is near. Our teaching of death education, in this case, focuses on philosophical reflection (e.g., what does death feel like?) and the transformation of life knowledge, or life wisdom, into practice. For us, personally, transformation of lifelong knowledge is integral to the negation and/or placation of one’s fear of death. For example, transformation of knowledge and personal understanding of Buddhist philosophy (Yeshe and Rinpoche, 1976; Xiao et al., 2017; Chattopadhyay, 2022), such as the tenet of ‘*saṃsāra’,* or the *endless cycle of birth, death, and rebirth*, may help a person to query and/or to consider the possibility that life continuously perpetuates and does not cease. This transformation, from our observation, is reflected by evidence, which showcases volunteers offering spiritual advice to those individuals who are dying and on the cusp of death (e.g., volunteers would make weekend visits and pray and recite the Buddhist sutras).

Transformation of knowledge or wisdom into practice, involving spiritual infusion (e.g., Buddhist philosophy) is hopeful and extraordinary, helping perhaps to alleviate one’s fear, angst, and distress about death and dying. In a similar vein, our teaching practice touches on another aspect of transformation, which is somewhat unconventional. In this case, we purport that transformation of personal understanding of ‘esoteric knowledge’ and/or ‘esoteric experiences’ (Phan et al., 2021a,b) into practice, where possible, may also help to ‘normalize’ a person’s perception and/or fear of death. One example of this is related to the notion, or concept, of what we term as ‘spiritual connectedness’. Spiritual connectedness, in this case, does not actually espouse and/or equate to a person’s perceived connectedness with some ‘divine being’ (e.g., God) in the physical world. Rather, in our context, spiritual connectedness entails the potentiality for a person, via means of meditation practice (Loden, 1996; Xiao et al., 2017; Chattopadhyay, 2022), traditional rituals (e.g., the ritual of ‘Guan Luo Yin’), etc. to ‘connect’ with ‘spiritual beings’ or ‘souls’ of loved ones who have moved on. The notion of spiritual connectedness is extremely fascinating as it delves into the unproven unknowns of this physical world, reflecting the important nature of esotericism or esoteric experience (Faivre, 1994; Pasi, 2015; Blake, 2020). Such thinking and/or rationalization contends that some form of life or that *the* ‘afterlife’ (Segal, 2004; Ellwood, 2010; Picone, 2012; Joaquin, 2019) in another non-physical realm is possible.

### 3.3. Summation for consideration

Overall, then, the preceding sections have introduced the teaching of life and death from the Taiwanese sociocultural-learning context. As a brief recap, we theorize that the subject of life and death may, in fact, support the theoretical scope of lifespan development (Peterson, 2004; Dacey et al., 2009; Sigelman et al., 2016). Our complementary teaching experiences and research inquiries, situated in different sociocultural-learning contexts (e.g., Australia) have led us to propose the following:

Teaching practice of life and death that emphasizes the importance of what we term as ‘theoretical infusion’, which involves the incorporation, or the infusion, of differing religious faiths, epistemological and cultural beliefs, customary practices, etc., which contends the infusion of spirituality in the teaching of life to help facilitate meaningful understanding of life and death experiences (e.g., Buddhist infusion or infusion of Buddhist philosophy). We contend that theoretical infusion for quality teaching of life and death may apply to other sociocultural-learning contexts – for example, the theoretical infusion of Islam (i.e., ‘Islamic infusion’) (Nasr, 1987/2008; Bonab et al., 2013), Christianity (Kourie and Ruthenberg, 2008; Knitter, 2009), Hinduism (Warrier, 2006; Srivastava and Barmola, 2013), Judaism (Jones, 2004), etc.

Teaching practice of life and death that emphasizes the importance of ‘active transformation’ of life knowledge, or life wisdom, into positive daily practice. Such positive practice (e.g., a teenager engages in a benevolent activity, reflecting her perceived sense of morality), we contend, would reflect one’s state of consciousness, love, and care for others in the community. Indeed, the theoretical premise of active transformation of life knowledge into positive practice reflects the important nexus between theory, research, and practicality (e.g., how would we transform a person’s extensive life wisdom of Buddhist spirituality or Islamic spirituality into effective practice for daily purposes?). Moreover, active transformation of life knowledge into practical relevance is significant, supporting our rationalization as to why the subject of lifespan development (Peterson, 2004; Dacey et al., 2009; Sigelman et al., 2016) may benefit from the inclusion of life and death.

## 4. Propose of a theoretical framework for consideration

Is there a universal theory or theoretical overview that one could use to approach the teaching and/or learning of life and death? One of our previous students, interestingly, provided a personal critique of life and death in his essay, which has indeed helped us to formulate our theoretical ideas. According to the student’s critique, subject contents pertaining to life and death are piecemealed and, more importantly, there is an absence of an overarching theory or theories that could serve to unify these independent strands of information. This assessment, we contend, is relatively accurate, especially when we compare life and death to other subject areas – for example, there is a theory known as *cognitive load* (Sweller, 2010; Sweller et al., 2011; Sweller, 2012) that may inform and assist educators to develop appropriate instructional designs for effective learning, and, likewise, there is a new, emerging theory known as *human optimization* (Phan et al., 2017, 2019, 2020a,b; Granero-Gallegos et al., 2023) that may account for a person’s achievement of ‘optimal best practice’ (Fraillon, 2004; Mascolo et al., 2010; Phan et al., 2016).

By all account, we acknowledge the complexity and/or the perplexity of the mentioned issue: that there is a need, perhaps, for us to consider some form of fluidity and/or cohesiveness, which would offer more meaningful understanding of the subject contents of life and death. Is there a *defining theoretical approach* and/or *theoretical framework* that is robust and/or inclusive, which we could use to teach and/or research life and death? Such discourse of having a logical theoretical framework, we contend, would ensure that information pertaining to life and/or death is sound, rational, and meaningful (e.g., why a relationship exists between two concepts).^3^ Moreover, from our point of view, such availability and/or existence of strong and/or consistent theoretical grounding could potentially help researchers with their future research inquiries. Our conceptualization then, as shown in Figure 1, considers the merging of different theoretical approaches – namely, in this case: the *Social viewpoint of life and death*, the *Psychological viewpoint of life and death*, and the *Philosophical viewpoint of life and death*.

### 4.1. Proposition for consideration

Our proposition, as Figure 1 shows, is innovative, highlighting the potential significance of the ‘merging’ or unification of three theoretical viewpoints. There are a number of defining reasons that may explain and/or justify why we choose to include and merge the social viewpoint, the psychological viewpoint, and the philosophical viewpoint within one overarching framework – for example:

▪ Recognizing the importance in *diversity* of theoretical viewpoints and, likewise, the expansion in personal understanding of life and death.
▪ Acknowledging *existing limitations* having just a philosophical approach to the teaching of life and death (Phan et al., 2021a,b; Seng and Lee, 2022).
▪ Considering the *effectiveness* of a wide range of practicalities for implementation, which could help improve life conditions and personal experiences.

We contend that a theoretical overview on its own (e.g., the philosophical viewpoint) is somewhat limited in terms of explanatory account. One major deterrence regarding this relates to the ‘restrictiveness’ in knowledge, ideas, reasonings, etc. that a person may acquire. For example, recapping our earlier discussions, a focus on acquiring life wisdom via philosophical understanding alone is rather confined. It is better and/or more appropriate, in this analysis, to consider a wider scope in coverage of subject contents, knowledge, and/or understanding of life and death. Merging the social viewpoint, the psychological viewpoint, and the philosophical viewpoint into one core theoretical framework is unique as it offers both contrasting and complementary viewpoints, which could help ‘generalize’ the subject of life and death to a wider, international audience for recognition. This premise contends, of course, the uniqueness of the following:

The *Philosophical Viewpoint*, which seeks to understand and appreciate the underlying meaning of life and death, via means of introspective reflection, higher-order consideration, and contemplation of personal experiences. As we described earlier, relevant research (Chen, 2009; Phan et al., 2021a,b; Seng and Lee, 2022) has considered one interesting element, namely, the fusion of spirituality (e.g., Buddhist spirituality) (Yeshe and Rinpoche, 1976; Chattopadhyay, 2022), which may contribute to a person’s life wisdom and help to ‘enlighten’ her understanding of life and death-related matters. For example, recapping a previous mentioning, spiritual understanding and/or spiritual connection to the Buddhist premise of *saṃsāra, or* the ‘*endless cycle of birth, death, and rebirth’, may bring comfort to those who are experiencing the onset of death* (i.e., the personal experience of dying and that death is near).

The *Psychological Viewpoint*, which Phan et al. (2020a,b) recently introduced, seeks to recognize the extent to which different psychological processes of learning could account for a person’s understanding of life and death. This viewpoint differs from the philosophical viewpoint and contends, interestingly, that existing psychological theories and theoretical tenets (e.g., the importance of positive psychology) (Seligman and Csíkszentmihályi, 2000; Seligman et al., 2009) may assist to explain a person’s perception, motivation, cognition, and/or behavior within the learning and/or non-learning context of life and death. For example, a person’s state of resilience (Almedom, 2005; Sanderson and Brewer, 2017; Chung et al., 2018) especially in times of negativities (e.g., a person battling a serious health issue) is advantageous, as it may help him to self-regulate his emotions, actions, behaviors, etc.

The *Social Viewpoint*, unlike the previous two viewpoints, considers the importance of the ‘social contexts’ of life and death, entailing a person and her relationship with the immediate social or physical environment. Social contexts or social milieus, constantly evolving with time, may serve to ‘subsume’ a person and make profound impacts on her perception, judgment, self-belief, and/or understanding of life and death-related matters. Importantly, however, this viewpoint places emphasis on daily practicality and proactive non-academic engagement in society, which may assist society, families, individuals, etc. to function effectively. For example, a person’s concerted attempts to connect with a social and/or religious network in the local community (Cohen, 2004; Churchill and Halverson, 2005; Mayes et al., 2009; Umberson and Montez, 2010; Sadiku et al., 2019) may encourage him to undertake a daily life practice (e.g., engaging in a group meditation), or a daily activity, for beneficial outcomes.

### 4.2. In summary: holistic understanding

Central to our conceptualization, as shown in Figure 1, is the rationalization that complementing with each other, the three theoretical viewpoints may help to facilitate one’s ‘holistic’ understanding of the subject contents of life and death. A singular theoretical viewpoint is rather restricted, confining knowledge and personal understanding of life and death to a narrow focus (e.g., simply a philosophical focus on life and death). By the same token, perhaps there is no overarching theory that may adequately explain the underlying nature of life and death. More appropriate therefore, from our aforementioned rationalization, is the integration, or unification, of comparative theoretical viewpoints – namely, the philosophical viewpoint, the psychological viewpoint, and the social viewpoint.

A unifying approach is innovative as it reflects the potential promotion, facilitation, and /or cultivation of different ideas, perspectives, and/or reasonings for the underlying nature of life and death. Moreover, we contend that a unifying approach may enable us to achieve the following:

- Instill and/or provide opportunities for *creativity* in teaching and curriculum development, which may entail the design and development of innovative comparative pedagogical practices for usage. For example, a cooperative learning exercise, using the JIGSAW method (Killen, 2009) in a class of 4–5 groups (e.g., each group consists of 3–4 students) may provide opportunities for students to learn about the complexity of life and death from each other (e.g., one group learns about the philosophical viewpoint and another group learns about the social viewpoint, etc.).
- Provide *guidance* (e.g., relevant information) to assist individuals with daily personal and/or social undertakings, which may promote, boost, and/or foster their differing understandings of the true meanings of life and death. For example, the social viewpoint of life and death may impart theoretical insights and inform students and others of the potential benefits (e.g., recognizing the advantage of experiencing Buddhist spirituality) of proactive engagement in daily life-related practice (e.g., engaging in a benevolent activity).
- Provide opportunities to *structure learning outcomes* of life and death that are diverse, interesting, and analytical (e.g., a comparative examination of the philosophical viewpoint versus the psychological viewpoint). Educators, for example, may wish to introduce various psychological theories of learning (e.g., Piaget’s (1932) *theory of personal cognition*) to help educate and/or advance student progress of the subject matter (e.g., a student’s appreciation of the learning and personal understanding of ‘morality’).
- Provide opportunities for *research advancement*, which may seek to explore different lines of inquiry for theoretical, empirical, and/or methodological contributions. For example, an inquiry into the positive effect(s) of a student’s weekly practice of helping a local temple or church may reflect the significance of the social viewpoint of life and death. In a similar vein, exploring a senior citizen’s psychological mindset as she responds to a maladaptive life condition may offer theoretical insights into the potency of the psychological viewpoint. A focus on personal experience of ‘spiritual transcendence’ (Hood and Morris, 1983; Rowe, 1997), likewise, may advance the study of the philosophical viewpoint of life and death.

## 5. Conceptualization of research inquiries for advancement

Aside from our teaching practice of life and death (e.g., the importance of theoretical infusion), we have also engaged in extensive research development of the subject area. Research undertakings may consist of different types – for example, conceptual inquiries, theoretical inquiries, empirical inquiries, philosophical inquiries, etc. To date, our research inquiries pertaining to the nature of life and death, collectively and individually, have been conceptual and philosophical. Philosophical inquiries emphasize the use of *philosophical psychology* and *philosophical reflection* (Thagard, 2014; Phan et al., 2021a,b), which consider the importance of personal intuition, logical reasoning and rationalization, and analysis of existing evidence. For example, our recent published article, using philosophical reflection as a theoretical basis (Phan et al., 2020a,b) detailed a conceptual framework, which situated life and death experiences within the framework of positive psychology (Seligman and Csíkszentmihályi, 2000; Seligman et al., 2009; Csíkszentmihályi, 2014). In a similar vein, we used philosophical reflection to seek clarity and understanding into the association between aspects of death and one’s experience of esoterism (Phan et al., 2021a,b).

Conceptual inquiries that involve philosophical reflection are innovative, as they encourage critical thinking, rationalization, and potential reconceptualization with logical reasoning (e.g., is there some form of life after death?). Moreover, research inquiries that depend on philosophical reflection are potentially open-ended with different and non-definitive solutions. In other words, unlike empirical inquiries that involve collection of primary-sourced data, philosophical inquiries are as such that they do not necessarily require scientific rigor and/or scientific validation. In the following section of the article, we explore three major inquiries – namely: the importance of *a person’s situated mindset*, testament of *transcendence* and *esoteric experiences*, and development of *appropriate methodological designs* for usage.

### 5.1. The importance of “situated mindset”

Our first proposed inquiry introduces a theoretical concept, which we term as ‘situated mindset’. Situated mindset, for us, is psychological and espouses a *person’s attitude*, *perception*, *connectedness*, *introspective reflection*, and *contemplation* of life and death-related matters. This considered viewpoint expands on previous theoretical tenets of ‘spiritual mindset’, which may espouse different religious affiliations – for example, ‘Islamic spiritual mindset’ (Nasr, 1987/2008; Bonab et al., 2013), ‘Christian spiritual mindset’ (Kourie and Ruthenberg, 2008; Van der Merwe, 2018), ‘Buddhist spiritual mindset’ (Thanissaro, 2015; Chattopadhyay, 2022), etc. A Buddhist spiritual mindset (Yeshe and Rinpoche, 1976; Xiao et al., 2017) for example, which may intimately associate with life and death education (Chen, 2009; Huang, 2014; Tsai et al., 2019), considers the perceived ‘connectedness’ and/or perceived feeling of ‘spiritual enlightenment’ experience (Yeshe and Rinpoche, 1976; Loden, 1996), or spiritual awakening (Chattopadhyay, 2022). One portal of communication or source of relevance, which may assist to facilitate a person’s Buddhist spiritual mindset is her proactive engagement in meditation (e.g., walking meditation).

**Figure 2:**
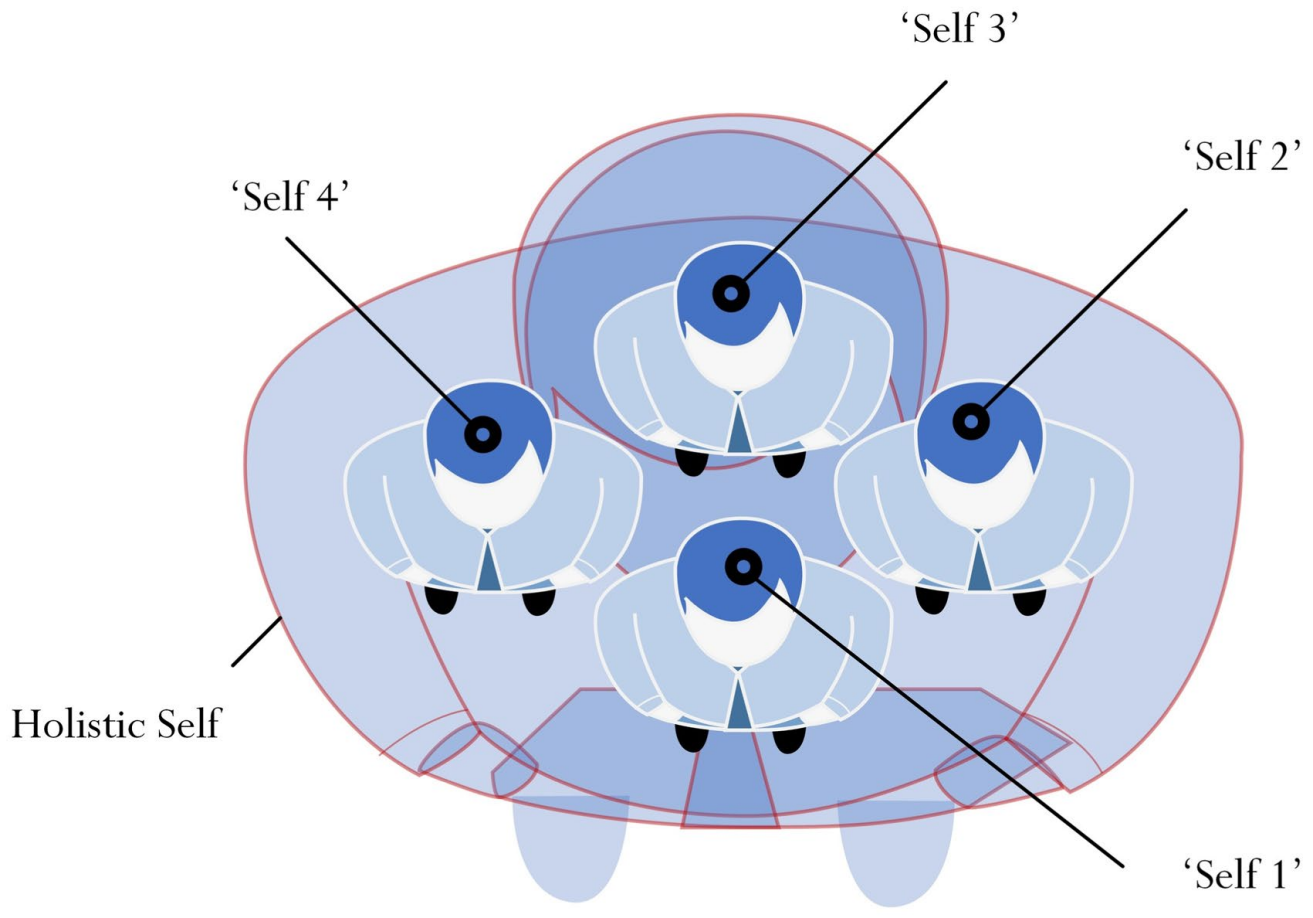
Proposed holistic self.

The study of spiritual mindset (e.g., Buddhist spiritual mindset) (Thanissaro, 2015; Chattopadhyay, 2022) is somewhat restricted as ‘spirituality’, in this case, does not fully and/or truly represent a person’s psychological mindset. In other words, spirituality alone does not define the totality of a person’s mindset. One underlying reason is that on a daily basis, perhaps, a person may not necessarily exhibit a spiritual mindset. Not every individual has the willingness, inclination, and/or interest to attain a ‘state of spirituality’ (Jones, 2004; Bonab et al., 2013; Srivastava and Barmola, 2013). This considered viewpoint contends, then, that a person’s psychological mindset does not necessarily have to associate with his spiritual experience and/or feeling of some religious faith (e.g., Christian spiritual mindset) (Kourie and Ruthenberg, 2008; Van der Merwe, 2018). Rather, from our point of view, it is more meaningful to include a wider scope, which may encompass a spiritual mindset and/or a non-spiritual mindset. A situated mindset, a theoretical concept that we recently conceptualized, is expansive in scope and may, in accordance with our rationalization, associate with the notion of a ‘holistic self’ (Phan et al., 2021a,b). In brief, the tenets of a holistic self suggest that rather than having a singular ‘self’, a person in fact has multiple selves (e.g., see Figure 2). In this sense, we conceptualize that a person’s holistic self is overarching, espousing and/or encompassing multiple, varying selves – for example: a person may have a ‘self’ of herself as a part-time university student (i.e., *Self 1*) and, in tandem to this self, a ‘self’ as an older sister to her younger siblings at home (i.e., *Self 2*), and a ‘self’ as a full-time bank employee at work (i.e., *Self 3*).

A person’s perceived holistic self, espousing multiple, varying selves (e.g., Self 1, Self 2, Self 3, etc.) may associate with and help to explain his situated mindset. Specifically, we rationalize that a person’s multiple selves (e.g., Self 1, Self 2, Self 3, etc.) give rise to his perceived feeling and experience of ‘multiple mindsets’, which may situate within and adhere to different contexts. Referring to our previous explanation, for example, a person’s mindset of being a university student (i.e., Self 1) is unique and differs from her mindset of being a bank employee (i.e., Self 3). In this sense, a person’s mindset, situating within a particular context, may be:

▪ *Personal* – that is, *personal mindset*, which may indicate a person’s consciousness of their contemplative thoughts of daily events.
▪ *Philosophical* – that is, *philosophical mindset*, which may indicate a person’s consciousness of their higher-order thoughtfulness, logical reasoning, and open-ended contemplation of different life matters.
▪ *Spiritual* – that is, *spiritual mindset*, which may indicate a person’s wishful thinking, contemplation, desire, etc. toward attainment of enlightenment, or spiritual awakening.
▪ *Cognitive* – that is, *cognitive mindset*, which may indicate a person’s consciousness of their cognitive reasoning, resolution (e.g., problem solving), and explanation of different academic and/or non-academic matters.

How does the theoretical concept of multiple mindsets operate or function? We conceptualize that as an analogy, multiple mindsets are like a coin, a person’s hand, and/or a Rubik’s Cube. For example, the constant changing of the palmar side and the dorsal side of a person’s hand may depict and/or indicate the ‘dynamics’ of his two contrasting mindsets at any moment in time. This analogy contends that personal mindset, encompassing comparable and contrasting types may ‘fluctuate’ or alter at any moment in time with reference to differing contexts (e.g., a work context versus an academic context). A context, in this case, is postulated to account for and to compel a person to adopt and manifest a particular mindset. The context of spirituality (e.g., a teenager goes to church), for instance, may serve to instill, facilitate, and/or result in a person’s ‘adoption’ and subsequent ‘manifestation’ of a Christian spiritual mindset (Kourie and Ruthenberg, 2008; Van der Merwe, 2018). In a similar vein, the context of academic learning of Algebra (Ngu and Yeung, 2013; Booth et al., 2015; Star et al., 2015) would give rise to a student’s adoption and subsequent manifestation of her cognitive mindset.

The proposed concept of situated mindset, as opposed to an exclusive spiritual mindset (Thanissaro, 2015; Chattopadhyay, 2022), is innovative for its emphasis on the notion of ‘contextualization’ or specificity – for example, what is the context at hand (e.g., academic learning versus employment versus community counseling, etc.)? In terms of research undertakings, then, it would be of interest to explore and validate our proposition of *the contextual nature of situated mindset* – for instance, a Catholic priests’ *spiritual mindset* during Holy Communion (i.e., the context of religion) versus a postgraduate student’s *philosophical mindset* during her doctorate confirmation (i.e., the context of academia). As such, we acknowledge that there is credence to advance research inquiries of conceptual, philosophical, and/or empirical nature. One notable empirical inquiry, in this case, may involve the collection of primary-sourced data, quantitative and/or qualitative, which could help to validate and affirm the aforementioned proposition: that a person, at any moment in time, could have multiple selves and multiple mindsets, depending on the specific context at hand has multiple selves and multiple mindsets. A qualitative approach (Esterberg, 2002; Maxwell, 2005; Lofland et al., 2006), involving the use of *in situ* observations and/or interviews, say, may help to elucidate and/or affirm the importance of multiple situated mindsets. What does a person indicate (e.g., attitude, perception, connectedness, introspective reflection, contemplation, etc.) in terms of situated mindset when she is engaging in her academic studies?

### 5.2. Transcendence and esoteric experience

Our second proposed inquiry is closely aligned to the theoretical concept of what is known as ‘transcendence’ or ‘spiritual transcendence’ (Hood and Morris, 1983; Long, 2000; Ruschmann, 2011). Some may contend that, perhaps, spiritual transcendence is something that is psychological, mythical, and/or non-scientific in nature. What is spiritual transcendence? Unlike other traditional psychological and/or achievement-related constructs (e.g., the concept of ‘optimal best practice’) (Fraillon, 2004; Mascolo et al., 2010; Phan and Ngu, 2021; Granero-Gallegos et al., 2023), spiritual transcendence is somewhat difficult for us to envisage, define, measure, and/or assess. Both Western and Eastern literatures do consider and have delved into the theoretical nature of transcendence (Maslow, 1969; Ruschmann, 2011; Gorelik and Shackelford, 2017; Llanos and Martínez Verduzco, 2022). For example, according to Levin and Steele (2005), transcendence is defined as subjective awareness and is something that is “beyond perception and beyond understanding” (p. 89). This broad definition, from our collective interpretation, is extremely abstract, connoting a person’s perceived ‘spiritual connectedness’ or union with God or some form of Divinity. In their overview, Levin and Steele (2005) equate transcendence, or spiritual transcendence, with other comparable terms [e.g., see Table 1 in Levin and Steele (2005), p. 90], such as ‘Clear Light’, ‘Cosmic Consciousness’, ‘God Experience’, ‘Mystic Experience’, ‘Satori’, ‘Peak Experience’, and ‘Subliminal Consciousness’. This description (e.g., equating spiritual transcendence as some form of ‘subliminal consciousness’) contends that a person’s experience of spiritual transcendence is something that extends beyond the realm of the physical body and its psychosocial boundaries.

Levin and Steele’s (2005) definition suggests that spiritual transcendence is extremely unique for its open-ended, abstract, anomalistic, and mystical nature. Does a person’s testament of spiritual transcendence entail and/or reflect her ‘relatedness’ to some ‘divine entity’? Does spiritual transcendence infer and/or espouse some form of consciousness with ‘something’ that is unexplained? Does personal experience of spiritual transcendence traverse the time–space dimension and involve something else altogether? Does spiritual transcendence intimately associate with deep, meaningful understanding of religious faiths (e.g., Islamic spiritual transcendence) (Nasr, 1987/2008; Bonab et al., 2013; Marzband et al., 2017)? Does spiritual transcendence reflect, indicate, and/or associate with a person’s perceived feeling of ‘self-enlightenment’? Is it possible for a person to assist another person to experience a perceived sense of spiritual transcendence (e.g., the notion of application or ‘practical transcendence’, where one is able to practice and help others to experience transcendence)? These purposive reflective questions, we contend, highlight the abstractness and open-ended and mystical nature of spiritual transcendence.

One interesting line of inquiry for consideration relates to the extent to which extensive practice of meditation (e.g., Buddhist meditation) (Hanh, 1976; Xiao et al., 2017; Wu et al., 2019) could, in fact, provide ‘grounding’ for a person to experience some form of spiritual transcendence. Moreover, from our personal experiences, we rationalize that a person’s ‘spiritual transcendence’ is intimately associated with her ‘esoteric experiences’. In-depth practice of meditation may give rise to the personal experience of ‘meditative insight’, which consists of the following: (i) an ‘out-of-body’ experience where a person’s ‘spiritual soul’ intimately connects with other esoteric entities (e.g., spiritual soul of a loved one who has passed away), (ii) a person’s ‘out-of-body’ experience, which traverses to another time–space realm, and (iii) a person’s ability to gauge into future events that have not yet happened and, likewise, concurrent events that she has no privy to. In essence, a person’s experience of meditative insight is partly esoteric and may, in this sense, reflect her experience of spiritual transcendence. In essence, in accordance with our proposition of life and death teaching, transcendence is something that expands and/or goes beyond one’s own state of consciousness and/or self-awareness of ‘closeness’ with some divine being, etc. (e.g., Buddha).

How do we empirically validate and/or affirm the existence of meditative insight, which may give rise to certain esoteric experiences (e.g., a person’s self-awareness of an event that is currently occurring at another sociocultural setting, or a person’s subconscious communication with a loved one who has moved on)? We acknowledge that personal experience of spiritual transcendence is subjective and individualized, reflecting uniqueness and variations among individuals. Even still, there is the question of personal experience in life – for example, does one have enough ‘insight’ and/or ‘experience’ to attain a state of spiritual transcendence? There are many unknowns that do not have definitive answers and, as such, anything is plausible in terms of validation and/or affirmation. Is it ever likely that we would be able to accurately measure, assess, and/or evaluate a person’s esoteric state of spiritual transcendence? One possibility, in this case, may involve personal recording or documentation of daily meditation practice (e.g., keeping a reflective journal with daily recording of thoughts, feelings, etc.), and whether such engagement could enable a person to experience unknown phenomena that may closely align with spiritual transcendence.

Seeking to validate the underlying nature of spiritual transcendence may also involve other possibilities, such as a person’s introspective reflection and evaluation of her ‘spiritual’ feelings, thoughts, emotions, etc. on a daily basis. One possible angle, in this case, may involve a person’s concerted effort to seek avenues and/or pathways, which could help him to experience a state of spiritual transcendence. In other words, is it possible for a person to experience the aforementioned attributes of spiritual transcendence (e.g., a person’s subconscious communication with a loved one who has moved on) without having to undertake meditation and/or any other mindfulness-related practice? We query, for example, whether the sharing of personal experiences of spiritual transcendence (e.g., via focus-group discussion) could help ‘enlighten’ a person’s self-awareness of his esoteric knowledge, understanding, etc.?

### 5.3. Methodological account for consideration

Our third proposed inquiry acknowledges the importance of what we term as ‘methodological consideration’ and/or ‘methodological appropriateness’ (Phan et al., 2019). Lifespan development research (e.g., the study of cognitive development), to date, has involved the use of quantitative, qualitative, and mixed methodological approaches. For example, researchers tend to rely on primary-sourced data, situated within experimental and/or non-experimental frameworks (e.g., a longitudinal approach that involves, say, the use of autoregressive structural equation modeling) to study children’s cognitive growths (e.g., Greene et al., 2004; Cury et al., 2006; Koskey et al., 2010). Having said this, we allude to the methodological difficulty that one would face when researching inquiries that are associated with subject matters pertaining to life and death education (Chen, 2009; Tsai et al., 2018; Phan et al., 2021a,b). How would we accurately and/or realistically measure, assess, and evaluate, say, the concept of spiritual transcendence? To date, to our knowledge, some researchers have used quantitative methodological designs – for example, Tsai and her Taiwanese colleagues (e.g., Tsai et al., 2019, 2020) have relied on experimental designs to investigate the impact of life education within the context of Medicine.

The proposed social viewpoint of life and death education, from our point of view, would require some alternative methodological designs. What would be an appropriate and/or adequate methodological design, which could assist researchers to measure, assess, and /or ‘capture’ the social practice of life and death? One of the co-authors of the present article, for example, has encouraged the use of personal diaries and/or journals to document a person’s reflective thoughts, feelings, emotions, etc. as she engages in different types of social discourse. Such practice of documenting daily actions and/or behaviors may encourage one to analyze, interpret, and/or make meaningful sense of his/her continuous life experiences. What have I done today that may assist me in my understanding of the importance of life practice? What do I need to engage in, socially and/or academically, in order to help me appreciate life? What have I learned so far from the local community in terms of spirituality?

In relation to the study of life and death (Tsai, 2008; Chen, 2009; Huang, 2014; Tsai et al., 2018), the notion of methodological alignment or methodological appropriateness is somewhat difficult to ascertain. One notable factor or reason for this uncertainty is related to the complex nature of the subject matter itself. We reason that a typical traditional methodological approach (e.g., the use of a Likert-scale survey) would not be sufficed and/or appropriate. One innovative methodological design for consideration, in this case, may involve using what is known as ‘meditative-reflective documentation’. Meditative-reflective documentation is a term that one of the co-authors of the present article has coined, reflecting the importance of one’s personal account of his insight and/or experience, which may intimately associate with the practice of Buddhist meditation (Yeshe and Rinpoche, 1976; Xiao et al., 2017). In brief, engagement in meditation (e.g., walking meditation) and experience of mindfulness (Thera, 1972; Hanh, 1976; Keng et al., 2011) may help and/or enable a person to introspectively analyze, reflect, and contemplate about any subject for that matter. As such, it is encouraged, as personal practice, to record one’s inner ‘meditative thoughts and insights’ for potential daily usage. Documentation of ‘meditative thoughts and/or insights’, in this case, may involve a person identifying and recording down specific keywords, drawings, and/or brief descriptions of her current thought, insight, emotion, feeling, etc. as he meditates – for example, “…. I feel pretty buoyant….,” “…. relaxation….,” “…. transience….,” “…. impermanence….,” etc. This personal approach, we contend, may also accentuate our theoretical premise of active transformation (i.e., to consider meditative thoughts and insights for daily relevance).

We contend that meditative-reflective documentation, aligning closely with the practice of meditation, is an innovative methodological approach for consideration. Moreover, from our point of view, meditative-reflective documentation may also help a person to seek deep, meaningful understanding of higher-order consciousness and subconsciousness of the mind. For example, individualized recall of keywords, descriptions, etc. (e.g., “…. transience….,” “…. impermanence….,” etc.) upon personal meditation practice may facilitate self-awareness of higher cognitive thoughts, and/or encourage a person to seek out different esoteric experiences of life, if possible (e.g., a person’s testament of her *subconscious* interpersonal communication with loved ones who have since passed on) (Phan et al., 2021a,b). Having said this, however, we do acknowledge that the perceived potency and/or effectiveness of meditative-reflective documentation requires further practice and investigative measures in order to determine whether our narrative is valid or not.

## 6. Final thoughts and summation

The present conceptual analysis article overall is significant for its attempts to advance the study of life and death (Chen, 2012; Huang, 2014; Tsai et al., 2020; Phan et al., 2020a,b) by situating it within the Taiwanese sociocultural-learning context. We hope that our theoretical overview and personal narrative may also help to advance the scope and subject contents of lifespan development (Peterson, 2004; Dacey et al., 2009; Sigelman et al., 2016). In this analysis, we contend that theoretical understanding of lifespan development may meliorate by taking into account the intricacies of life and death education. Our teaching development and research undertakings to date, individually and collectively, have enabled us to conceptualize and to engage in several important discourses – for example, our emphasis on the notion of ‘active transformation’ of life knowledge, or life wisdom, and our pedagogical strategy involving the practice of ‘theoretical infusion’, which may help to facilitate effective teaching and learning of life and death.

Despite extensive progress to date, we acknowledge that one interesting deficiency or inadequacy is that there is no definitive and/or overarching theoretical approach that may help to facilitate effective teaching and appreciation of life and death education. It is our collective interest, in this sense, to develop an overarching theoretical framework that may situate and apply to different sociocultural-learning contexts. As such, we have conceptualized a theoretical framework that is unifying, consisting of three distinct viewpoints: the social viewpoint of life and death, the philosophical viewpoint of life and death, and the psychological viewpoint of life and death. We firmly believe that such advocation of having comparative theoretical viewpoints for teaching and research (e.g., to consider a combination of social viewpoint + philosophical viewpoint) may, in fact, help to widen the scope and coverage of the subject matter.

Finally, using philosophical reflection as a theoretical approach, we conceptualize a few notable research inquiries for advancement – namely, in this case: the notion of situated mindset, the potential for a person to indicate transcendence and esoteric experiences, and the design and/or development of appropriate methodological approaches to investigate life and death-related matters. The three inquiries, we contend, are innovative and reflect our collective interest to seek new frontiers in research development. For example, we rationalize that research inquiries involving non-traditional methodological designs (e.g., the use of meditative-reflective documentation) may help to expand on formal knowledge of different facets of life and death and, hence, lifespan development – for example, the use of philosophical analysis, contemplative insight, and logical reasoning to seek deep, meaningful understanding of a person’s situated mindset, the extent to which a person could actually traverse from the present physical time-space realm to some unknown, etc.

## Author contributions

HP and BN were responsible for the literature search and writeup of this article. HP, BN, S-CC, and C-SH contributed equally to the conceptualization and theoretical contribution of the article. All authors contributed to the article and approved the submitted version.

## Conflict of interest

The authors declare that the research was conducted in the absence of any commercial or financial relationships that could be construed as a potential conflict of interest.

## Publisher’s note

All claims expressed in this article are solely those of the authors and do not necessarily represent those of their affiliated organizations, or those of the publisher, the editors and the reviewers. Any product that may be evaluated in this article, or claim that may be made by its manufacturer, is not guaranteed or endorsed by the publisher.

## Footnotes

^1^ https://www.aitsl.edu.au/standards/graduate

^2^ https://laveti.org/blogs/news/before-during-and-after

^3^ https://eric.ed.gov/?id=ED491769#:~:text=Theories%20are%20vital%3A%20They%20guide,sound%20basis%20for%20practical%20action

## References

[ref1] Abel D. C. (1989). Freud on instinct and morality: on instinct and morality. New York, NY: University of New York Press.

[ref2] Almedom A. M. (2005). Resilience, hardiness, sense of coherence, and posttraumatic growth: all paths leading to “light at the end of the tunnel”? J. Loss Trauma 10, 253–265. doi: 10.1080/15325020590928216

[ref3] Bandura A. (2018). Toward a psychology of human agency: pathways and reflections. Perspect. Psychol. Sci. 13, 130–136. doi: 10.1177/1745691617699280 29592657

[ref4] Bazalgette L. Rahilly T. Trevelyan G. (2015). Achieving emotional well-being for looked after children: A whole system approach. Wales: NSPCC.

[ref5] Blake A. (2020). Understanding what is esoteric. Correspondences 8, 1–25.

[ref6] Bonab B. G. Miner M. Proctor M.-T. (2013). Attachment to god in Islamic spirituality. J. Muslim Mental Health 7, 77–104. doi: 10.3998/jmmh.10381607.0007.205

[ref7] Booth J. L. Oyer M. H. Paré-Blagoev E. J. Elliot A. J. Barbieri C. Augustine A.. (2015). Learning algebra by example in real-world classrooms. J. Res. Educ. Effect. 8, 530–551. doi: 10.1080/19345747.2015.1055636

[ref8] Carrera E. (2009). Lovesickness and the therapy of desire: Aquinas, cancionero poetry, and Teresa of Avila's 'Muero porque no muero. Bulletin Hispanic Stud. 86, 729–742. doi: 10.1353/bhs.0.0098

[ref9] Chapple H. S. Bouton B. L. Chow A. Y. M. Gilbert K. R. Kosminsky P. Moore J.. (2017). The body of knowledge in thanatology: an outline. Death Stud. 41, 118–125. doi: 10.1080/07481187.2016.1231000, PMID: 27611636

[ref10] Chattopadhyay M. (2022). Contemplation: its cultivation and culmination through the Buddhist glasses. Front. Psychol. 12:281. doi: 10.3389/fpsyg.2021.800281, PMID: 35449693 PMC9017814

[ref11] Chen S.-C. (2001). Research on the experiment and reflection of the education of awareness: taking the course of 'wareness and Life' of Hua fan University as an example. Hua Fan J. 7, 74–89.

[ref12] Chen S.-C. (2009). The fusion of life and health-spiritual education. Natl. Educ. 50, 7–13.

[ref13] Chen S.-C. (2012). Oriental humanities, mindfulness and life education. Life education symposium, Taipei City, National Taipei University of Education.

[ref14] Chen S.-C. (2013). Overview and reflection on the 20-year National Education Life Education Curriculum. Natl. Educ. 53, 1–6.

[ref15] Chen W. W. Ho H. Z. (2012). The relation between perceived parental involvement and academic achievement: the roles of Taiwanese students' academic beliefs and filial piety. Int. J. Psychol. 47, 315–324. doi: 10.1080/00207594.2011.630004, PMID: 22288600

[ref16] Chow S. S.-Y. Chu M. H.-T. (2007). The impact of filial piety and parental involvement on academic achievement motivation in Chinese secondary school students. Asian J. Counsel. 14, 91–124.

[ref17] Chung J. Lam K. Ho K. Cheung A. Ho L. Gibson F.. (2018). Relationships among resilience, self-esteem, and depressive symptoms in Chinese adolescents. J. Health Psychol. 25, 2396–2405. doi: 10.1177/1359105318800159 30229681

[ref18] Churchill E. Halverson C. (2005). Social networks and social networking. IEEE Int. Comput. 9, 14–19. doi: 10.1109/MIC.2005.103

[ref19] Ciarrochi J. Parker P. Kashdan T. B. Heaven P. C. L. Barkus E. (2015). Hope and emotional well-being: a six-year study to distinguish antecedents, correlates, and consequences. J. Posit. Psychol. 10, 520–532. doi: 10.1080/17439760.2015.1015154

[ref20] Cohen S. (2004). Social relationships and health. Am. Psychol. 59, 676–684. doi: 10.1037/0003-066X.59.8.676 15554821

[ref21] Cowan P. A. Longer J. Heavenrich J. Nathanson M. (1969). Social learning and Piaget's cognitive theory of moral development. J. Pers. Soc. Psychol. 11, 261–274. doi: 10.1037/h0027000 5784266

[ref22] Csíkszentmihályi M. (2014). Toward a psychology of optimal experience. Flow and the foundations of positive psychology, Berlin: Springer: 209–226.

[ref23] Cury F. Elliot A. J. Da Fonseca D. Moller A. C. (2006). The social-cognitive model of achievement motivation and the 2 x 2 achievement goal framework. J. Pers. Soc. Psychol. 90, 666–679. doi: 10.1037/0022-3514.90.4.666, PMID: 16649862

[ref24] Dacey J. Travers J. Fiore L. (2009). Human development across the lifespan. New York, NY: McGraw Hill.

[ref25] Dorough S. (2011). “Moral development” in Encyclopedia of child behavior and development. eds. Goldstein S. Naglieri J. A. (Springer US: Boston, MA), 967–970.

[ref26] Ellwood R. (2010). Tales of lights and shadows: Mythology of the afterlife. London, Bloomsbury Publishing Plc.

[ref27] Esterberg K. G. (2002). Qualitative methods in social research. New York, NY: McGraw Hill.

[ref28] Faivre A. (1994). Access to Western esotericism. Albany, New York: SUNY Press.

[ref29] Fraillon J. (2004). Measuring student well-being in the context of Australian schooling: Discussion paper. Carlton South, VIC: The Australian Council for Research.

[ref30] Frankl V. E. (1984). Man’s search for meaning: An introduction to Logotherapy. New York, Simon & Schuster.

[ref31] Gorelik G. Shackelford T. K. (2017). What is transcendence, how did it evolve, and is it beneficial? Religion Brain Behav. 7, 361–365. doi: 10.1080/2153599X.2016.1249928

[ref32] Granero-Gallegos A. Phan H. P. Ngu B. H. (2023). Advancing the study of levels of best practice pre-service teacher education students from Spain: associations with both positive and negative achievement-related experiences. PLoS One 18:e0287916. doi: 10.1371/journal.pone.0287916, PMID: 37390102 PMC10313018

[ref33] Greene B. A. Miller R. B. Crowson H. M. Duke B. L. Akey K. L. (2004). Predicting high school students' cognitive engagement and achievement: contributions of classroom perceptions and motivation. Contemp. Educ. Psychol. 29, 462–482. doi: 10.1016/j.cedpsych.2004.01.006

[ref34] Hanh T. N. (1976). Miracle of mindfulness. Boston, Beacon.

[ref35] Hasmath R. (2014). Social development: theory and practice, by James Midgley. J. Dev. Stud. 50, 1321–1323. doi: 10.1080/00220388.2014.936661

[ref36] Havens T. (2013). Confucianism as humanism. CLA J. 1, 33–41.

[ref37] Hood R. W. Morris R. J. (1983). Toward a theory of death transcendence. J. Sci. Study Relig. 22, 353–365. doi: 10.2307/1385773

[ref38] Huang S.-Y. (1993). Health promotion and health education. Taipei City, Normal University Shuyuan.

[ref39] Huang J. (2014). New orientation of life education in the 21st century: Spiritual awakening, classic study and environmental education. Proceedings of the ninth life education conference, Taipei City, Taiwan Life Education Society.

[ref40] Joaquin J. J. (2019). Hell, heaven, neither, or both: the afterlife and Sider’s puzzle. Sophia 58, 401–408. doi: 10.1007/s11841-018-0682-5

[ref41] Jones D. H. (1966). Freud's theory of moral conscience. Philosophy 41, 34–57. doi: 10.1017/S0031819100066134

[ref42] Jones M. (2004). Judaism, spirituality, and disability. J. Religion Disabil. Health 8, 55–88. doi: 10.1300/J095v08n01_06

[ref43] Keng S.-L. Smoski M. J. Robins C. J. (2011). Effects of mindfulness on psychological health: a review of empirical studies. Clin. Psychol. Rev. 31, 1041–1056. doi: 10.1016/j.cpr.2011.04.006, PMID: 21802619 PMC3679190

[ref44] Killen R. (2009). Effective teaching strategies: Lesson from research and practice. South Melbourne, VIC: Cengage Learning.

[ref45] Knitter P. F. (2009). Without Buddha I could not be a Christian. Croydon, Oneworld Publications.

[ref46] Koskey K. L. K. Karabenick S. A. Woolley M. E. Bonney C. R. Dever B. V. (2010). Cognitive validity of students’ self-reports of classroom mastery goal structure: what students are thinking and why it matters. Contemp. Educ. Psychol. 35, 254–263. doi: 10.1016/j.cedpsych.2010.05.004

[ref47] Kourie C. Ruthenberg T. J. (2008). Contemporary Christian spirituality: a worldly embodiment. Koers 73, 303–322. doi: 10.4102/koers.v73i2.164

[ref48] Kristiansen M. Sheikh A. (2012). Understanding faith considerations when caring for bereaved Muslims. J. R. Soc. Med. 105, 513–517. doi: 10.1258/jrsm.2012.120284 23288085 PMC3536508

[ref49] Lei L. Lu Y. Zhao H. Tan J. Luo Y. (2022). Construction of life-and-death education contents for the elderly: a Delphi study. BMC Public Health 22:802. doi: 10.1186/s12889-022-13197-7, PMID: 35449042 PMC9022733

[ref50] Levin J. Steele L. (2005). The transcendent experience: conceptual, theoretical, and epidemiologic perspective. Explore 1, 89–101. doi: 10.1016/j.explore.2004.12.002, PMID: 16781509

[ref51] Lickona T. (1991) in Moral development in the elementary school classroom. Moral behavior and development: Vol. 3. Application. eds. Kurtines W. M. Gewirtz J. L. (Erllbaum: Hillsdale, NJ)

[ref52] Llanos L. F. Martínez Verduzco L. (2022). From self-transcendence to collective transcendence: in search of the order of hierarchies in Maslow’s transcendence. Front. Psychol. 13:787591. doi: 10.3389/fpsyg.2022.787591 35401301 PMC8988189

[ref53] Loden G. A. T. (1996). Meditations on the path to enlightenment. Melbourne, VIC: Tushita Publications.

[ref54] Lofland J. Snow D. Anderson L. Lofland L. H. (2006). Analyzing social settings: A guide to qualitative observation and analysis. Belmont, CA: Thomson.

[ref55] Long J. (2000). Spirituality and the idea of transcendence. Int. J. Child. Spiritual. 5, 147–161. doi: 10.1080/713670913

[ref56] Marzband R. Moallemi M. Darabinia M. (2017). Spiritual nutrition from the Islamic point of view. J. Islamic Stud. Cult. 5, 33–39. doi: 10.15640/jisc.v5n2a4

[ref57] Mascolo M. F. College M. Fischer K. W. (2010) in The dynamic development of thinking, feeling and acting over the lifespan. Biology, cognition and methods across the life-span. ed. Overton W. F. (Hoboken, NJ: Wiley), 1.

[ref58] Masel E. K. Schur S. Watzke H. H. (2012). Life is uncertain. Death is certain. Buddhism and palliative care. J. Pain Sympt. Manag. 44, 307–312. doi: 10.1016/j.jpainsymman.2012.02.018 22871512

[ref59] Maslow A. H. (1969). Various meanings of transcendence. J. Transpers. Psychol. 1, 56–66.

[ref60] Maxwell J. A. (2005). Qualitative research design: An interactive approach. Thousand Oaks, CA, Sage Publications.

[ref61] Mayes L. Magidson J. Lejeuz C. Nicholls S. (2009). Social relationships as primary rewards: the neurobiology of attachment. Handbook of developmental social neuroscience. Haan M. De Gunnar M.. New York, The Guilford Press: 342–377.

[ref62] Meagher D. J. Balk D. E., (2013). Handbook of thanatology. London: Routledge.

[ref63] Ministry of Education Taiwan (2018). Life education intermediate plan of the Ministry of Education (2018/8/1–2022/7/31). Taipei City, Ministry of Education.

[ref64] Nasr S. H., (1987/2008). Islamic spirituality: Foundations. New York, Routledge.

[ref65] Ngu B. H. Yeung A. S. (2013). Algebra word problem solving approaches in a chemistry context: equation worked examples versus text editing. J. Math. Behav. 32, 197–208. doi: 10.1016/j.jmathb.2013.02.003

[ref66] Parsell C. Eggins E. Marston G. (2016). Human agency and social work research: a systematic search and synthesis of social work literature. Br. J. Soc. Work 47, bcv145–bcv255. doi: 10.1093/bjsw/bcv145

[ref67] Pasi M. (2015). “Esoteric experiences and critical ethnocentrism” in Religion: Perspectives from the Engelsberg seminar 2014. eds. Almqvist K. Linklater A. (Stockholm: son Johnsons Foundation), 131–142.

[ref68] Patanella D. (2011). “Piaget’s theory of moral development” in Encyclopedia of child behavior and development. eds. Goldstein S. Naglieri J. A. (Boston, MA: Springer), 1109–1111.

[ref69] Peterson C. (2004). Looking forward through the lifespan. Frenchs Forest, NSW: Pearson.

[ref70] Phan H. P. Ngu B. H. (2019). Teaching, learning and psychology. Docklands, Melbourne, Oxford University Press.

[ref71] Phan H. P. Ngu B. H. (2021). Optimization: an attempt to establish empirical evidence for theoretical and practical purposes. Eur. J. Psychol. Educ. 36, 453–475. doi: 10.1007/s10212-020-00484-3

[ref72] Phan H. P. Ngu B. H. Chen S.-C. Wu L. Lin W.-W. Hsu C.-S. (2020a). Introducing the study of life and death education to support the importance of positive psychology: an integrated model of philosophical beliefs, religious faith, and spirituality. Front. Psychol. 11, 1–16. doi: 10.3389/fpsyg.2020.580186 33117246 PMC7578223

[ref73] Phan H. P. Ngu B. H. Chen S.-C. Wu L. Shih J.-H. Shi S.-Y. (2021a). Life, death, and spirituality: a conceptual analysis for educational research development. Heliyon 7:e06971. doi: 10.1016/j.heliyon.2021.e06971, PMID: 34036188 PMC8138599

[ref74] Phan H. P. Ngu B. H. McQueen K. (2020b). Future time perspective and the achievement of optimal best. Front. Psychol. 11, 1–13. doi: 10.3389/fpsyg.2020.01037 32670133 PMC7326102

[ref75] Phan H. P. Ngu B. H. White M. (2021b). Introducing ‘holistic psychology’ for life qualities: a theoretical model for consideration. Heliyon 7:e05843. doi: 10.1016/j.heliyon.2020.e05843, PMID: 33474507 PMC7803644

[ref76] Phan H. P. Ngu B. H. Williams A. (2016). Introducing the concept of optimal best: theoretical and methodological contributions. Education 136, 312–322.

[ref77] Phan H. P. Ngu B. H. Yeung A. S. (2017). Achieving optimal best: instructional efficiency and the use of cognitive load theory in mathematical problem solving. Educ. Psychol. Rev. 29, 667–692. doi: 10.1007/s10648-016-9373-3

[ref78] Phan H. P. Ngu B. H. Yeung A. S. (2019). Optimization: in-depth examination and proposition. Front. Psychol. 10, 1–16. doi: 10.3389/fpsyg.2019.01398 31275210 PMC6593188

[ref79] Piaget J. (1932). The moral judgment of a child. Glencoe, IL: The Free Press.

[ref80] Picone M. (2012). Suicide and the afterlife: popular religion and the standardisation of ‘culture’ in Japan. Cult. Med. Psychiatry 36, 391–408. doi: 10.1007/s11013-012-9261-3, PMID: 22549663

[ref81] Prude A. (2019). “Death in Tibetan Buddhism” in Death and dying: an exercise in comparative philosophy of religion. eds. Knepper T. D. Bregman L. Gottschalk M., vol. 2 (Cham: Springer International Publishing), 125–142.

[ref82] Rahul V. C. (2018). Taiwan – its religion and beliefs. Int. J. Adv. Res. Dev. 3, 42–45.

[ref83] Reif S. C. Reif S. C. Lehnardt A. Bar-Levav A. (2014). Death in Jewish life: Burial and mourning customs among Jews of Europe and nearby communities. Berlin, De Gruyter.

[ref84] Rowe W. L. (1997). “Death and transcendence” in Religion without transcendence? Claremont studies in the philosophy of religion. eds. Phillips D. Z. Tessin T. (London, UK: Palgrave Macmillan)

[ref85] Ruschmann E. (2011). Transcending towards transcendence. Implicit Religion 14, 421–432. doi: 10.1558/imre.v14i4.421

[ref86] Sadiku M. Omotoso A. Musa S. (2019). Social networking. Int. J. Trend Sci. Res. Dev. 3, 126–128. doi: 10.31142/ijtsrd21657

[ref87] Sammons P. Sylva K. Melhuish E. Siraj-Blatchford I. Taggart B. Hunt S.. (2008). “Influences on children's cognitive and social development in year 6” in Effective pre-school and primary education 3–11 project (EPPE 3–11). Research brief, vol. 10 (London: Department for Children, schools and families)

[ref88] Sanderson B. Brewer M. (2017). What do we know about student resilience in health professional education? A scoping review of the literature. Nurse Educ. Today 58, 65–71. doi: 10.1016/j.nedt.2017.07.018, PMID: 28898767

[ref89] Saso M. R. (1970). The Taoist tradition in Taiwan. China Q. 41, 83–102.

[ref90] Segal A. F. (2004). Life after death: A history of the afterlife in Western religion. New York, Doubleday.

[ref91] Seligman M. Csíkszentmihályi M. (2000). Positive psychology: an introduction. Am. Psychol. 55, 5–14. doi: 10.1037/0003-066X.55.1.5 11392865

[ref92] Seligman M. Ernst R. M. Gillham J. Reivich K. Linkins M. (2009). Positive education: positive psychology and classroom interventions. Oxf. Rev. Educ. 35, 293–311. doi: 10.1080/03054980902934563

[ref93] Seng H. Z. Lee P. W. (2022). Death education in Malaysia: from challenges to implementation. Int. J. Pract. Teach. Learn. 2, 1–8.

[ref94] Sheng Yen M. (2010). The dharma drum lineage of Chan Buddhism: Inheriting the past and inspiring the future. Taipei City, Taiwan, The Sheng Yen Education Foundation.

[ref95] Sigelman C. K. Rider E. A. George-Walker L. D. (2016). Lifespan human development. South Melbourne, VIC: Cengage Learning Australia.

[ref96] Son N. D. Nga G. B. (2019). “Death and dying: Belief, fear and ritual in Vietnamese culture” in Death across cultures: Death and dying in non-Western cultures. eds. Selin H. Rakoff R. M. (Cham: Springer International Publishing), 75–82.

[ref97] Spice L. M. (2011). The effect of induced happiness levels on academic performance. Undergraduate Honors Thesis. Indianapolis: Butler University.

[ref98] Srivastava S. K. Barmola K. C. (2013). Rituals in Hinduism as related to spirituality. Indian J. Positive Psychol. 4, 87–95.

[ref99] Stanger N. Kavussanu M. Boardley I. D. Ring C. (2013). The influence of moral disengagement and negative emotion on antisocial sport behavior. Sport Exerc. Perform. Psychol. 2, 117–129. doi: 10.1037/a0030585

[ref100] Star J. R. Pollack C. Durkin K. Rittle-Johnson B. Lynch K.. (2015). Learning from comparison in algebra. Contemp. Educ. Psychol. 40, 41–54. doi: 10.1016/j.cedpsych.2014.05.005

[ref101] Sternberg R. Glück J., Eds. (2019). The Cambridge handbook of wisdom. Cambridge: Cambridge University Press.

[ref102] Sternberg R. Glück J. (2021). Wisdom: The psychology of wise thoughts, words, and deeds. Cambridge, Cambridge University Press.

[ref103] Sweller J. (2010). Element interactivity and intrinsic, extraneous, and germane cognitive load. Educ. Psychol. Rev. 22, 123–138. doi: 10.1007/s10648-010-9128-5

[ref104] Sweller J. (2012). “Human cognitive architecture: Why some instructional procedures work and others do not” in APA Educational Psychology handbook. eds. Harris K. Graham S. Urdan T., vol. 1 (Washington DC: American Psychological Association), 295–325.

[ref105] Sweller J. Ayres P. Kalyuga S. (2011). Cognitive load theory. New York, Springer.

[ref106] Tabbodi M. Rahgozar H. Abadi M. M. M. (2015). The relationship between happiness and academic achievements. Europ. J. Nat. Soc. Sci. 4, 241–246.

[ref107] Thagard P. (2014). The self as a system of multilevel interacting mechanisms. Philos. Psychol. 27, 145–163. doi: 10.1080/09515089.2012.725715

[ref108] Thanissaro P. N. (2015). The spirituality of Buddhist teens: religious/spiritual experiences and their associated triggers, attributes and attitudes. Int. J. Children's Spiritual. 20, 218–232. doi: 10.1080/1364436X.2015.1118680

[ref109] Thera N. (1972). The power of mindfulness. San Francisco, CA, Unity Press.

[ref110] Tsai Y. (2008). Life and philosophy of life: definition and clarification. Comment. Philos. Natl. Taiwan Univ. 35, 155–190.

[ref111] Tsai F. J. Chen C. Y. Yeh G. L. Hu Y. J. Tseng C. C. Chen S. C. (2018). Nursing students’ relationships among meaning in life, well-being, and positive beliefs a cross-sectional survey study. Medicine (United States) 97:e12914. doi: 10.1097/MD.0000000000012914 PMC6211886 30335023

[ref112] Tsai F. J. Hu Y. J. Chen C. Y. Yeh G. L. Tseng C. C. Chen S. C. (2019). Simulated directed-learning in life-education intervention on the meaning of life, positive beliefs, and well-being among nursing students: a quasi-experimental study. Medicine 98:e16330. doi: 10.1097/MD.0000000000016330, PMID: 31277181 PMC6635261

[ref113] Tsai F. J. Hu Y. J. Yeh G. L. Chen C. Y. Tseng C. C. Chen S. C. (2020). The effectiveness of a health promotion intervention on the meaning of life, positive beliefs, and well-being among undergraduate nursing students: one-group experimental study. Medicine 99:e19470. doi: 10.1097/MD.0000000000019470, PMID: 32150107 PMC7478399

[ref114] Umberson D. Montez J. K. (2010). Social relationships and health: a flashpoint for health policy. J, Health Social Behav. 51, 54–66. doi: 10.1177/0022146510383501 PMC3150158 20943583

[ref115] Van der Merwe D. (2018). The characterisation of the spiritual Christian: in conversation with god according to 1 Corinthians 2. HTS Teologiese Studies/Theol. Stud. 74:a4968

[ref116] Warrier M. (2006). Faith guides for higher education: A guide to Hinduism. Oxford: Alden Group Limited.

[ref117] Wu R. Liu L.-L. Zhu H. Su W.-J. Cao Z.-Y. Zhong S.-Y.. (2019). Brief mindfulness meditation improves emotion processing. Front. Neurosci. 13:1074. doi: 10.3389/fnins.2019.01074, PMID: 31649501 PMC6795685

[ref118] Xiao Q. Yue C. He W. Yu J.-Y. (2017). The mindful self: a mindfulness-enlightened self-view. Front. Psychol. 8:1752. doi: 10.3389/fpsyg.2017.01752 29081754 PMC5645519

[ref119] Yao X. (2000). An introduction to Confucianism. Cambridge: Cambridge University Press.

[ref120] Yeshe L. Rinpoche L. Z. (1976). Wisdom energy: Basic Buddhist teachings. Somerville, MA: Wisdom Publications.

